# The Effect of TNF-*α* on CHD and the Relationship between TNF-*α* Antagonist and CHD in Rheumatoid Arthritis: A Systematic Review

**DOI:** 10.1155/2022/6192053

**Published:** 2022-08-24

**Authors:** Yezhou Qian, Menghui Mao, Feige Nian

**Affiliations:** ^1^Department of Cardiology, The First Hospital of Jiaxing, The Affiliated Hospital of Jiaxing University, Jiaxing, China; ^2^Department of Rheumatology, The First Hospital of Jiaxing, The Affiliated Hospital of Jiaxing University, Jiaxing, China

## Abstract

Tumor necrosis factor-alpha (TNF-*α*) plays an important role in coronary heart disease (CHD), a chronic inflammatory process. Meanwhile, this pro-inflammatory factor is also involved in the pathogenesis of autoimmune diseases such as rheumatoid arthritis (RA). Patients with RA correspond to a higher risk of CHD. TNF-*α* antagonist, one of the main treatments for RA, may reduce the risk of CHD in patients with RA. This review summarizes the pathogenesis of TNF-*α* in CHD and discusses the relationship between TNF-*α* antagonist and CHD in patients with RA.

## 1. Introduction

In recent years, the incidence of coronary heart disease (CHD) has increased year by year, and its mortality rate has already surpassed that of cancer [1]. The underlying mechanism of CHD is atherosclerosis, and the oxidative modification of low-density lipoprotein cholesterol (LDL-C) is the main cause of plaque formation [2]. At present, the occurrence and development of CHD are generally considered as a chronic inflammatory process and tumor necrosis factor-alpha (TNF-*α*) has significant effects on the development of CHD [3]. It has been shown that TNF-*α* is capable of damaging endothelium function, enhancing the uptake of ox-LDL by macrophages, promoting angiogenesis, and triggering the clinical coronary events [4–7]. As a pro-inflammatory cytokine, TNF-*α* is central to the inflammatory process of autoimmune diseases such as rheumatoid arthritis (RA) [8]. Patients with RA have higher risk of CHD [9]. Meanwhile, TNF-*α* antagonists have been shown to be effective in controlling inflammatory activity and functional impairment in RA [10]. There is accumulating evidence indicating that the use of TNF-*α* antagonists is associated with a reduced risk of cardiovascular events in patients with RA [11–19]. However, other studies have found TNF-*α* antagonists to be associated with no change or increased cardiovascular risk [20, 21]. In this review, we summarize the recent advances of TNF-*α* in the pathogenesis of CHD and discuss the impact of TNF-*α* antagonists on CHD in patients with RA.

## 2. Overview of TNF-*α*

TNF-*α* is originally discovered during 1975 that could kill mouse tumor cells, which is why we call it “tumor necrosis factor” [22]. It belongs to the TNF superfamily of proteins consisting of 157 amino acids and is mainly generated by activated macrophages, T-lymphocytes, and natural killer cells [23, 24], but several subsequent studies have shown that it is also produced by nonimmune cells such as endothelial cells, adipocytes, neurons, and myocardial cell [25–28]. TNF-*α* exists in two forms: transmembrane (tmTNF-*α*) or soluble TNF-*α* (sTNF-*α*) [29]. tmTNF-*α* is expressed on the surface of activated lymphocytes, macrophages, and other cell types, and when processed by TNF-*α*-converting enzyme, it is released as the sTNF-*α* [30, 31]. The biological activity of TNF-*α* is achieved through two receptors: TNF-*α* receptor 1 (TNFR1) and TNF-*α* receptor 2 (TNFR2) [32]. TNFR1 is expressed in most nucleated cells, and it is fully activated by both tmTNF-*α* and sTNF-*α* [33]. TNFR2 is expressed mainly in immune cells but also in myocardial cell and is primarily activated by tmTNF-*α* in the context of cell-to-cell interactions [34, 35]. TNF-*α* is involved in many pathophysiological processes, such as inflammation, immunity, cell proliferation, apoptosis, and lipid metabolism [36–38]. Abnormal secretion of TNF-*α* leads to various diseases, such as RA [39], inflammatory bowel disease [40], spondylarthritis [41], psoriasis [42], noninfectious uveitis [43], and CHD [44].

## 3. TNF-*α* and CHD

### 3.1. TNF-*α* and Endothelial Cell

In vascular homeostasis, vascular endothelial cells act as a barrier [45]. Disruption of the barrier leads to inflammatory cell invasion, which contributes to a variety of vascular diseases, including atherosclerosis [46]. Research suggests that TNF-*α* disrupts the intercellular connections of endothelial cells and enhances vascular permeability [4, 47]. A large number of studies reveal that endothelial inflammation plays an important role in the progression of atherosclerosis [48]. TNF-*α* activates endothelial cells and induces monocytes/macrophages to express cytokines and chemokines, which may lead to the progression of atherosclerosis [48]. In the development of atherosclerosis, endothelial cell apoptosis plays an important role in the regulation [4]. TNF-*α* induces endothelial cell apoptosis by upregulating autophagy, which is inhibited by arachidonic acid [49]. In addition to apoptosis, endothelial cell senescence is positively associated with the development of atherosclerosis [50], and exposure to TNF-*α* promotes premature endothelial senescence [51].

### 3.2. TNF-*α* and Foam Cell

In the early stages of atherosclerosis, monocytes migrate to the intima of coronary artery and differentiate into macrophages [52]. When oxidized low-density lipoprotein (ox-LDL) intake exceeds the metabolic capacity of macrophages, macrophages transform into foam cells [43]. Foam cells are involved in fatty streak formation, a hallmark of the early stages of atherosclerosis [53]. The study confirms that TNF-*α* promotes monocyte adhesion to endothelial cells, which is effectively blocked by adalimumab [54]. ox-LDL induces oxidative stress and increases TNF-*α* secretion by macrophages via reducing the inhibition effect of miR-491-5p on matrix metalloproteinase 9 [55]. Meanwhile, TNF-*α* enhances the uptake of ox-LDL by macrophages in a concentration-dependent manner [5]. There is a vicious circle here, where TNF-*α* promotes the uptake of ox-LDL by macrophages, and this in turn increases the release of TNF-*α*. In addition, the formation of foam cells is also associated with impaired cholesterol efflux from macrophages [56], TNF-*α* has been shown to reduce cholesterol efflux by suppressing the expression of adenosine triphosphate (ATP)-binding membrane cassette transporter A1 (ABCA1) and liver *X* receptor-*α*, and infliximab exerts atheroprotective effect by eliminating the reduction in foam cells induced by TNF-*α* [57].

### 3.3. TNF-*α* and Angiogenesis

Angiogenesis is an essential process in a variety of physiological and pathological conditions, including atherosclerosis and rheumatoid arthritis [58]. It contains the differentiation, proliferation, migration, and maturation of endothelial cells [59]. Vascular endothelial growth factor (VEGF) is an important mediator of angiogenesis [60], and VEGF augments vascular endothelial cell proliferation, migration, and survival [60]. TNF-*α* promotes VEGF expression and angiogenesis [6]. Besides, TNF-*α* mediates the expression of chemerin in human coronary endothelial cells under hypoxia and promotes the early process of angiogenesis [61]. Studies have shown that chemerin stimulates angiogenesis both in vitro and in vivo to a similar extent as that of VEGF [62]. Chen et al. also find that the angiogenic function of TNF-*α* is significantly enhanced with the overexpression of angiopoietins 1 and 2 [63]. In endothelial cell inflammatory responses, angiopoietin sensitizes endothelial cells to TNF-*α* [64].

### 3.4. TNF-*α* and Vascular Smooth Muscle Cell

**Figure 1:**
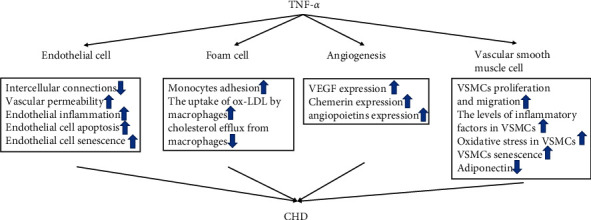
Specific mechanism of TNF-*α* in CHD.

Abnormal migration, extracellular matrix synthesis, and proliferation of vascular smooth muscle cells (VSMCs) contribute to the formation of atherosclerotic plaque [65]. TNF-*α* causes VSMC proliferation and migration through multiple pathways [66–71]. It contains upregulated expression of lncRNA HIX003209, miR-21, lncRNA CAMK2D-associated transcript-1, miR-375-3p, Raf-1/MAPK-dependent manner, and the help of matrix metalloproteinase 2. Vascular calcification is associated with CHD [72]. Adiponectin, secreted by adipocytes, protects VSMCs from calcification induced by beta-glycerophosphate by inhibiting the JAK kinase 2/signal transduction and activator transcription 3 signaling pathway and downregulating the expression of the transcription factor osterix [73]. TNF-*α* impairs adiponectin multimerization, consequently decreasing adiponectin secretion by altered disulfide bond modification in endoplasmic reticulum [74]. Inflammation is implicated in atherosclerosis along with the accumulation of leukocytes and inflammatory mediators such as interleukin (IL)-1*β* and IL-6, and TNF-*α* increases the levels of inflammatory factors in VSMCs [75]. Abnormal oxidative stress in VSMCs plays an important role in the occurrence and development of vascular remodeling and promotes the development of atherosclerosis [76]. This oxidative stress in VSMCs is induced by TNF-*α,* and overexpression of 17*β*-estradiol abolishes this pathological process [77]. Atherosclerosis, an age-related cardiovascular disease, is associated with cellular senescence and senescence-associated secretory phenotype in VSMCs [78]. TNF-*α* is one of the main inflammatory signaling molecules involved in the senescence of VSMCs by inducing the activity of senescence-associated *β*-galactosidase (SA-*β*-gal) and telomerase [79]. The specific mechanism of TNF-*α* in CHD is shown in Figure 1.

### 3.5. TNF-*α* and Myocardial Infarction

After myocardial infarction, a large amount of TNF-*α* is produced by ischemia and hypoxia-activated cardiomyocytes and local mononuclear macrophages [80, 81]. At the same time, the concentrations of TNFR1 and TNFR2 are also significantly increased [82]. STEMI patients with significantly elevated levels of TNF-*α* are more likely to have subsequent ischemic events, HF, and all cardiovascular events [7]. Since TNF-*α* induces the release of soluble TNF-*α* receptors 1 and 2 (sTNFR1 and sTNFR2) into the circulation in STEMI patients, these patients with high circulating sTNFR1 or sTNFR2 are at high risk of adverse clinical events [83]. Furthermore, TNF-*α* is also involved in adverse remodeling after myocardial infarction [84].

## 4. TNF-*α* Antagonist and CHD in Patients with RA

### 4.1. TNF-*α* Antagonist in RA

TNF-*α* antagonists are developed following the discovery that TNF-*α* plays a role in the pathophysiology of RA [85]. Five different drugs based on blocking TNF-*α* are available: infliximab, adalimumab, etanercept, golimumab, and certolizumab pegol [86]. Over the past few decades, numerous clinical trials have been conducted on these compounds, which have shown excellent and comparable efficacy in improving clinical, functional, and radiological disease outcomes in patients with RA [87]. As the most frequently used biologics in RA [88], in addition to some rare but serious systemic side effects, TNF-*α* antagonists may also exert pharmacological effects beyond the treatment of RA [14, 89, 90].

### 4.2. RA and CHD

Compared with the general population, patients with RA have 1.5–2 times increased risk of myocardial infarction and CHD [9,91]. The study has shown that the risk of cardiovascular disease (CVD) may increase before RA is diagnosed [92]. Patients with RA are often accompanied by disability, but CVD is the leading cause of death [93]. Studies show that the risk of CVD associated with RA is similar to diabetes [94]. Notably, the atherosclerotic burden in RA correlates with the disease severity at baseline [95] and RA activity over time may contribute to the risk of CVD [96]. Systemic inflammation is an important contributor to increased cardiovascular risk in patients with RA [20, 97]. TNF-*α* plays an important role in this pathological process [98]. Patients with RA are systemically predisposed to high levels of TNF-*α* [99]. It is generally accepted that RA and atherosclerosis are autoinflammatory diseases involving multiple inflammatory cytokines, with many common genetic predispositions and environmental factors [100].

### 4.3. TNF-*α* Antagonist and Endothelial Cell

Impaired endothelial cell function has been demonstrated in patients with RA and may contribute to the progression of atherosclerosis in these patients [101, 102]. As a cornerstone of RA treatment, a study shows that TNF-*α* antagonists improve endothelial function in patients with RA [103]. As a TNF-*α* antagonist, adalimumab is one of the leading therapies for RA [104]. It limits the inflammation of vascular by preventing endothelial activation, endothelial monocyte adhesion, and endothelial leakage [54]. Certolizumab pegol, another TNF-*α* antagonist, has also been shown to attenuate the pro-inflammatory state of endothelial cells [105]. Another study on certolizumab pegol indicates that leukocyte adhesion and angiogenesis induced by TNF-*α* could be suppressed by certolizumab pegol [106]. Endothelial progenitor cells have the ability to differentiate into endothelial cells in situ and limit the formation of atherosclerotic plaque, and short-term treatment of RA with TNF-*α* antagonists is associated with an increase in circulating endothelial progenitor cells [107]. Elevated levels of some soluble adhesion molecules, such as vascular cell adhesion molecule-1, are associated with endothelial dysfunction and the development of atherosclerosis [108], and administration with a TNF-*α* antagonist, certolizumab pegol, also has a positive effect on reducing the expression of some adhesion molecules [109].

### 4.4. TNF-*α* Antagonist and Lipid Profile

Risk factors for CHD include elevated plasma low-density lipoprotein cholesterol (LDL-C) and decreased high-density lipoprotein cholesterol (HDL-C) [110]. Dyslipidemia, considered as a secondary impact of chronic inflammatory state, has been found in patients with RA [111]. Treatment with TNF-*α* antagonists induces elevated serum HDL-C levels in patients with RA [112, 113]. This may be due to the fact that during the inflammatory process, the expression of cytokines such as TNF-*α* reduces the level of circulating HDL-C and TNF-*α* antagonists have the ability to control disease activity [114]. Results from other literature studies are conflicting, they have not found that TNF-*α* antagonists affect the levels of HDL-C in patients with RA [115–117], and this may be attributable to the differences in study populations, study duration, therapeutic drugs, and lack of adjustment for covariables such as age and comorbidities. A study by Hassan et al. follows up for 104 weeks, and the result shows no significant changes in the HDL-C and LDL-C values following the use of TNF-*α* antagonist [118]. Notably, LDL-C decreases significantly throughout the study in patients treated with statins. Concomitant treatment with TNF-*α* antagonist and statins may reduce the cardiovascular risk in patients with RA in addition to treating the inflammatory component. TNF-*α* antagonist not only affects the concentration of HDL-C but also enhances the antioxidant capacity of HDL-C and improves its anti-atherosclerotic ability [119]. This may explain that in patients with RA, the incidence of cardiovascular events decreases without higher HDL-C concentrations when treated with TNF-*α* antagonists.

### 4.5. TNF-*α* Antagonist and CHD Events

In patients with RA, the increased burden of CHD, particularly acute myocardial infarction (AMI), is independent of traditional CVD risk factors, and it is partly attributable to chronic systemic inflammation [120]. The use of TNF-*α* antagonists in RA reduces the risk of CHD events, such as MI, cardiac death, and unstable angina, and these risks are further reduced with long-term use [14], but another study shows that compared with receiving conventional modified antirheumatic drugs, the AMI rate is not reduced in RA treated with TNF-*α* antagonists, and reduction in this risk presupposes a response to TNF-*α* antagonists [17]. This finding supports that suppression of inflammation may reduce cardiovascular risk. Circulating TNFR1 levels are associated with mortality risk in AMI [121]. TNFR2 plays an important role in myocardial survival and homeostasis by suppressing apoptosis and necroptosis [122]. Cardioprotective effects of TNF-*α* antagonists may be related to the inhibition of TNFR1 [123]. However, inhibition of TNFR2, a cardioprotective receptor, by TNF-*α* antagonists exceeds that of TNFR1, resulting in increased cardiovascular morbidity [99]. The contrast in the risk of CVD can be explained by the difference in doses administered, causing different degrees of inhibition in TNFR2. Besides, the reduction in the risk of CHD events by TNF-*α* antagonists may be associated with the inappropriate use of glucocorticoids in control patients [19]. The risk of hypertension, diabetes, weight gain, and metabolic syndrome are increased with the use of glucocorticoids [124–126]. Meanwhile, these complications increase the risk of CHD in patients with RA.

### 4.6. TNF-*α* Antagonist and Others

RA is an independent risk factor for the development of CHD [127], and this can be explained by a prothrombotic state with abnormalities in the coagulation, fibrinolytic systems, and platelet reactivity [128]. The study provides evidence that the inhibition of fibrinolysis in patients with RA is reduced by TNF-*α* antagonist [129]. This helps to reduce the risk of thrombosis systematically. In patients with RA, traditional CVD risk factors such as diabetes, hypertension, and hyperlipidemia do not fully account for the increase in atherosclerosis [130]. Insulin resistance increases in patients with RA and is associated with accelerated coronary atherosclerosis [131], and TNF-*α* antagonists have been shown to improve insulin sensitivity and reduce insulin resistance in patients with RA [132]. Coronary artery calcification is part of the atherosclerotic process and is proportional to the risk of cardiovascular events [133]. It is worth noting that coronary calcium scores are significantly elevated in RA with inflammatory anemia [134], and TNF-*α* antagonists improve inflammatory anemia in patients with RA [135].

## 5. Conclusion and Perspective

In conclusion, patients with RA have a significant increase in CHD morbidity and mortality than patients without RA. Inflammation is the common link between CHD and RA. TNF-*α* is involved as an important inflammatory cytokine. Growing evidence suggests that there is a protective association between TNF-*α* antagonists and CHD in RA.

From endothelial cell dysfunction to myocardial infarction, TNF-*α* is widely involved in the occurrence and development of CHD [46, 136, 137]. As a chronic inflammatory disease, TNF-*α*-involved vascular inflammation plays an important role in the progression of CHD [138]. In local inflammation, TNF-*α* is released by inflammatory cells, endothelial cells, and cardiomyocytes [23–28]. It then mediates endothelial dysfunction, foam cell formation, angiogenesis, smooth muscle proliferation, and thrombosis [6, 46, 56, 66].

The incidence of CVD is significantly elevated in patients with RA, and it is the leading cause of death in patients with RA [139]. In addition to improving clinical, functional, and radiological disease outcomes in patients with RA [87], TNF-*α* antagonists, the most frequently used biologics in RA [88], improve endothelial function [103], lipid metabolism [112, 113], and the risk of CHD events [14].

Finally, as biologics become more prominent in the treatment of RA, future research should focus on determining whether TNF-*α* antagonists may directly exert cardioprotective effects through some unknown mechanism. Considering the pathogenic role of TNF-*α* in various stages of CHD, TNF-*α* antagonists may play a positive role in the treatment of CHD in the future.

## Data Availability

This is a review, with no underlying data.
